# Skin malformation assessment by triple wavelength imaging

**DOI:** 10.3389/fmed.2026.1851235

**Published:** 2026-07-01

**Authors:** Janis Spigulis, Ilze Irbe, Uldis Rubins, Edgars Kviesis-Kipge, Marta Skrastina

**Affiliations:** Biophotonics Laboratory, Faculty of Science and Technology, Institute of Atomic Physics and Spectroscopy, University of Latvia, Riga, Latvia

**Keywords:** avoided motion artefacts, diagnostic spectral imaging, multi-laser illumination, prototype validation, skin

## Abstract

This invited paper reviews recent results achieved by snapshot triple wavelength imaging of skin, a method ensuring high spectral and spatial resolution within sub-second imaging time, avoiding motion artefacts in the spectral image sets. Designs of the developed diagnostic equipment and the related image processing solutions are discussed along with outcomes of their clinical validation on volunteers with various skin malformations, including cancers. The initial contact technology for single skin lesion spectral imaging in the visible range has been extended to remote full body or large area multi-lesion image acquisition and analysis, including also near-infrared skin images. The potential of triple wavelength imaging for implementation in routine dermatological practice is discussed.

## 1 Introduction

Timely detection and accurate diagnosis of skin tumors and tumor-suspicious lesions remain clinically challenging and socially significant tasks. Among these conditions malignant melanoma is considered the most aggressive and dangerous form due to its high metastatic potential and diverse subtypes including acral lentiginous melanoma, melanoma *in situ*, nodular melanoma, and superficial spreading melanoma (1). A widely used approach is color imaging of skin lesions by a dermatoscope, smartphone camera or some specialized device(s), with following clinical conclusions based on doctor’s experience and/or artificial intelligence (AI) support (2). Still, specific details of skin pathology regions are better revealed in spectral images related to narrow wavelength bands. For example, hyperspectral imaging techniques can provide rich information on cancerous skin lesions (3). However, wide implementation of this technique is limited by costly equipment and complicated procedures for extraction clinically useful diagnostic criteria from the captured spectral image set.

Commercial color cameras detect images in three spectral bands – red (R, ~600–700 nm), green (G, ~500–600 nm), and blue (B, ~400–500 nm). Output data of RGB image sensor allows separating the red, green and blue broadband spectral images (which is impossible for human vision). Imaging with RGB cameras also allow selecting much narrower spectral bands if the images are captured under spectrally specific illumination that comprises only three discrete spectral lines, each of them positioned within one detection band of the camera (4). The triple wavelength imaging (3WI) concept is based on the target’s ability to reflect only these spectral components that are involved in the illumination spectrum. Under single spectral line illumination, the detected image represents a spectral image related only to the narrow wavelength interval covered by this spectral line. In the case of triple spectral line illumination, the RGB image dataset comprises information on three narrowband spectral images which can be extracted from this dataset by means of a relatively simple algorithm (4).

If compared to traditional multispectral imaging approaches where time-demanding sequential changes of narrowband optical filters or light emitting diodes (LEDs) for illumination are used (5), the 3WI technique offers single snapshot acquisition of all three spectral images, so excluding any movement artefacts in the spectral image sets. Another advantage is improved spectral selectivity thanks to extremely narrow spectral imaging bandwidth, typically <0.1 nm (6).

Contemporary RGB lasers are well suited light sources for 3WI, with condition that uniform illumination of the target simultaneously by all three emitted spectral lines is ensured. Besides, precise knowledge of the camera’s relative spectral sensitivities at all three exploited wavelengths is needed, along with means for reducing grainy laser speckle artefacts in the spectral images. These issues were addressed in our patented solutions (7–9), which allowed assembling several unique 3WI prototype devices for diagnostic imaging of skin and validating them on volunteering patients with diagnosed skin malformations, including malignancies.

Over the recent decade, several improvements of this originally developed technology have been proposed and reported in engineering/photonics-related conferences and journals (10–13). As different information sources are commonly used by the technical and medical communities, this Special Issue paper introduces novel technology to active dermatologists, cosmetologists and related clinical staff, emphasizing its potential diagnostic advantages. The scope and purpose of the article is somewhat non-traditional; its main goal is explaining details how to obtain snapshot spectral information with reduced motion artefacts using RGB camera acquisition under specific triple spectral line illumination, and how the newly obtained clinical data may facilitate advanced skin diagnostics. Based on our previous publications, a brief review of the 3WI technical aspects and its potential applications for non-invasive human skin assessment is presented below. In addition to the already published material, design of the remote imaging device is described in more detail, and a deeper parameter-based and AI-based analysis of the clinical results is performed, including calculations of quantitative diagnostic criteria like sensitivity and specificity. Advantages and limitations of the developed 3WI technology in comparison with common skin imaging methods are discussed.

## 2 Prototype devices for triple wavelength imaging of skin

### 2.1 Smartphone add-on 3WI device

**Figure 1:**
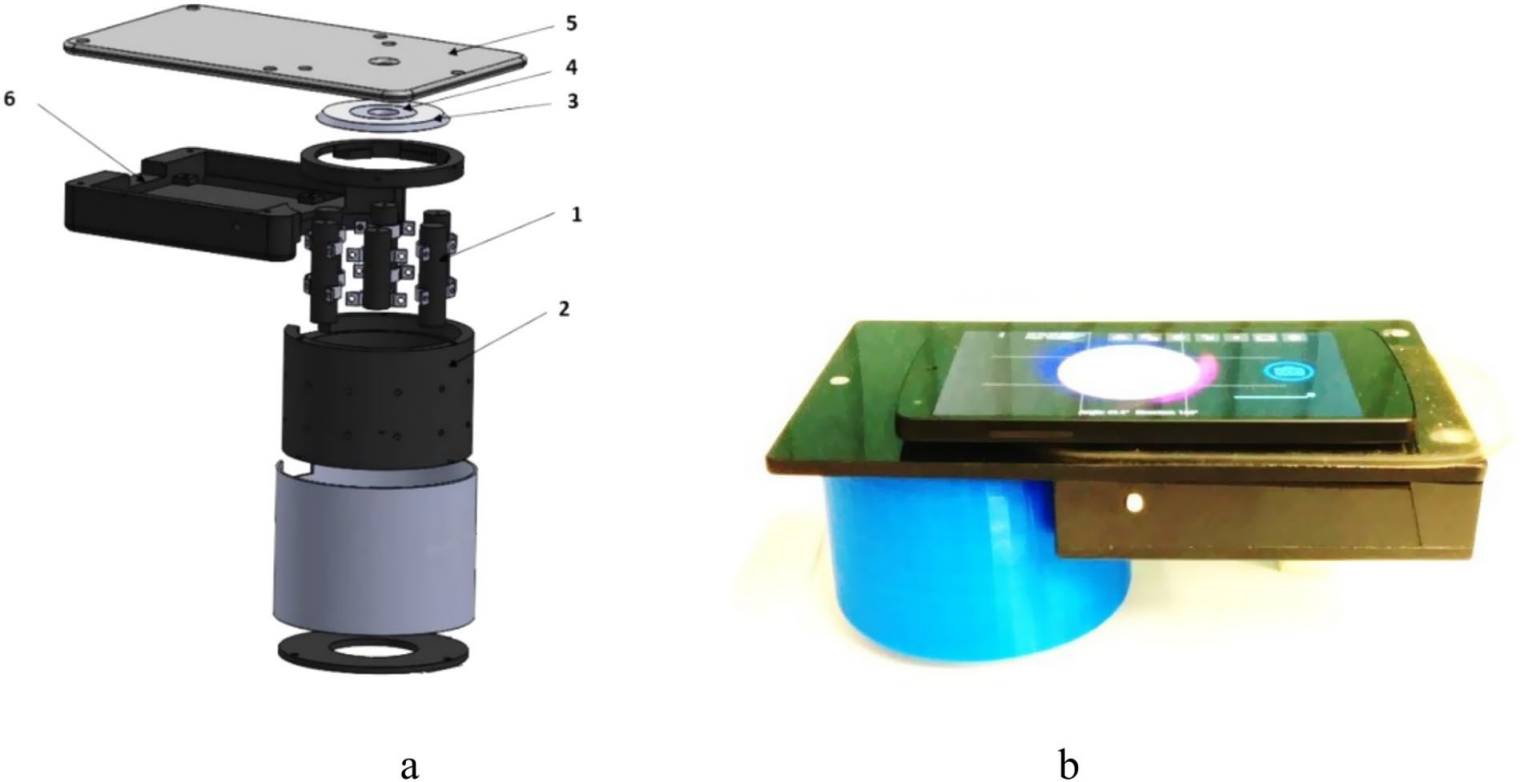
3D model of the 3WI illumination unit **(a)** and the mobile prototype with smartphone attached on it **(b)**. 1 – laser modules (3 pairs, 448–532-659 nm), 2 – shielding cylinder, 3 – collector of laser beams, 4 – flat ring-shaped diffuser of laser light, 5 – sticky platform for the smartphone, 6 – electronics compartment (11).

Following our first laboratory tabletop setup (10), a compact smartphone-compatible prototype device for single skin lesion assessment has been developed (11). Figure 1 presents its design details (a) and outlook of an operating prototype with a smartphone on it (b). The illumination wavelengths 448 nm, 532 nm and 659 nm are emitted by three pairs of compact 20 mW power laser modules (1), mounted on the internal wall of a hollow 3D-printed plastic shielding cylinder (2). The round bottom opening of this cylinder (diameter 40 mm) is in contact with skin and forms the field of view for the smartphone camera. The 45-degree conical reflecting edge of transparent disc (3) turns all laser beams towards its center, while the internal ring-shaped flat diffuser (4) scatters the three-wavelength laser light down to the skin. The *Google Nexus5* smartphone comprising 8 Mpx image sensor *SONY IMX179* with known (provided by manufacturer) RGB-sensitivities is placed on a flat sticky platform (5) with round window for the smartphone rear camera, co-aligned with the internal opening of the diffuser (4). Spatial resolution of the smartphone 3WI device is ~10 microns per pixel, overcoming that for conventional dermatoscopes (~50…75 microns). To avoid detection of skin surface-reflected light by the smartphone camera, cross-polarizer system is used: the round camera window is covered by a film polarizer, and another film with orthogonal direction of polarization covers from bottom the diffuser (4). The sticky platform design allows nearly any models of smartphones or tablet computers to be used for image capturing, independently of their dimensions.

Two rechargeable batteries and laser power supply circuits are placed in the 3D-printed plastic compartment (6) below platform (5). Target illumination intensity is adjusted to ensure linearity of photodetection by the smartphone camera. Its automatic settings are switched off by the *AZ Camera* software, so the R-, G- and B-outputs of the image sensor respond accordingly to the spectral sensitivities of its three detection bands.

**Figure 2:**
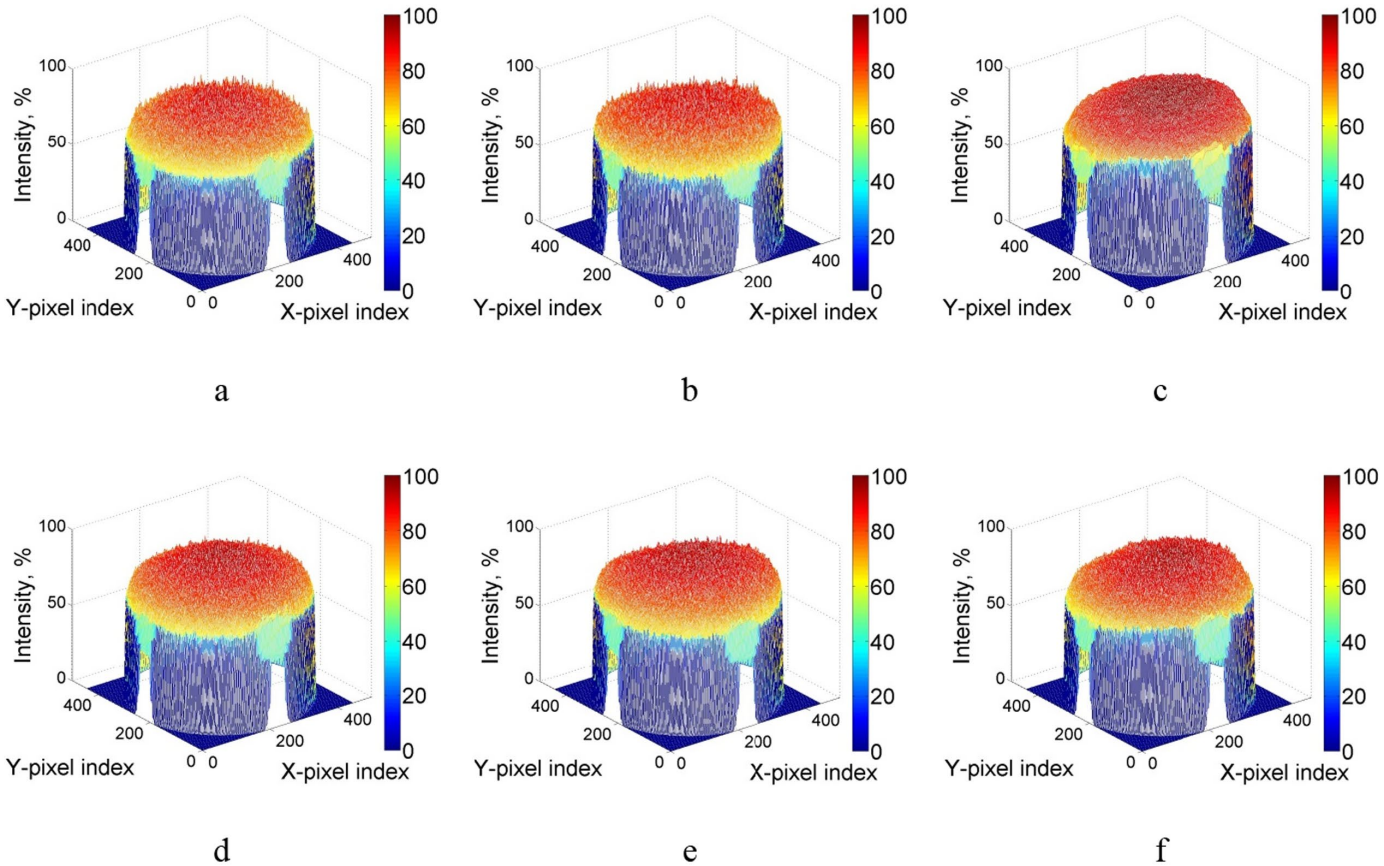
Uniformity of white paper illumination at different illumination-detection combinations: **(a)** – R-image at 659 nm illumination; **(b)** – G-image at 532 nm illumination; **(c)** – B-image at 448 nm illumination; **(d,e,f)** – R-, G- and B-images at simultaneous 3-wavelength illumination. Color scale represents relative intensities (11).

The above-described optical design ensures uniform illumination of the target. It was initially checked at all combinations of illumination wavelengths (448 nm, 532 nm, 659 nm) and image sensor detection channels (R, G, B), using white paper as the target. Figure 2 illustrates the obtained illumination intensity distributions, detected at separated RGB output channels in two cases – using only one wavelength and simultaneously all three wavelengths for illumination.

### 2.2 Remote whole-body 3WI device

A prototype for remote imaging of whole-body or large area skin under triple laser line illumination has been developed, as well. For distant skin illumination, RGB laser-coupled side-emitting optical fibers are used, while the images are captured by a high-resolution color camera (12, 13).

**Figure 3:**
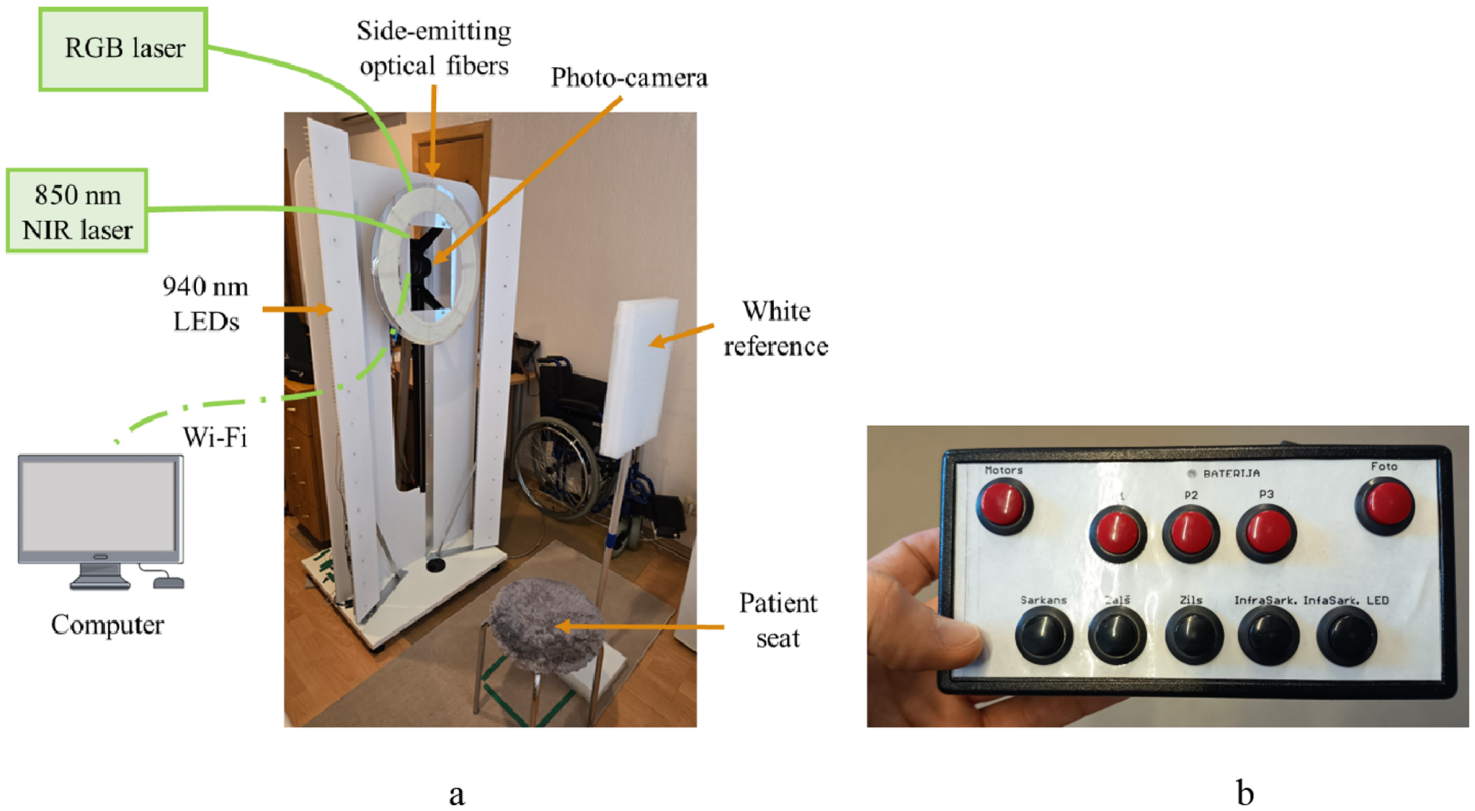
The prototype for full body remote triple-wavelength imaging of large skin areas (13) **(a)** and a switchboard for its wireless operation **(b)**.

Photo of the device is presented in Figure 3a. It consists of an illumination module and a high-resolution imaging module. Illumination module enables simultaneous or sequential emission of spectral lines at wavelengths 450 nm, 520 nm and 638 nm from a 3 W RGB laser and/or 850 nm from a 4 W laser (both from *HangZhou NaKu Technology Co., Ltd*., CN). The lasers are SMA-coupled to two spirals of silica core side-emitting optical fibers with distal end reflectors that ensure uniform lateral emission at three laser wavelengths simultaneously; total lengths of the fibers are 60 m and 30 m, respectively. Both fiber spirals are glued to a mirror plate which also serves as a structural holder.

The side-emitting fiber loops ensure uniform illumination of the working plane 70 cm apart, with measured intensity deviations not exceeding +/− 10%. Illumination power density at the working distance reaches 22 μW/cm^2^ for 450 nm, 17 μW/cm^2^ for 520 nm, 15 μW/cm^2^ for 638 nm, and 34 μW/cm^2^ for 850 nm, i.e., well below the ~200 mW/cm^2^ laser safety limit for skin (14). NIR illumination is additionally provided by two side panels with 940 nm LEDs (SMB1N-940DS-02, *Roithner Lasertechnik GmbH*, DE) emitting in total up to 30 W, with illumination power density below 0.2 mW/cm^2^ at the working conditions. In this paper, only 850 nm laser line images will represent the NIR spectral range, as they appeared nearly identical to those at 940 nm.

Inside the fiber spirals, a 61-megapixel *Sony Alpha 7R IV* full-frame color camera with switched-off automatic settings and removed infrared-blocking filter is placed. The field-of-view for recorded images is 700 × 1,050 mm, with spatial resolution ~0.1 mm per pixel. Both the laser-coupled fiber spirals and the camera form a single unit, mounted on a 1-meter linear rail and driven vertically by a stepper motor. The height of camera with illumination fibers can be varied between 0.5 m and 1.5 m, which allows non-contact imaging of patient’s large skin areas at any location on his/her body. For calibration purposes, images of a white reference (ColorChecker White Balance, *X-Rite*, *Inc*., United States), placed at the working distance, are always taken in parallel with skin images. To avoid skin illumination by ambient light, the imaging procedure is performed either in a dark room or in a light-shielding tent (12).

Device is operated wirelessly from a specially assembled switchboard (Figure 3b) which allows switching on/off each illumination wavelength independently, as well as using pre-installed combinations (buttons L, P2, P3). Besides, the switchboard enables remote capturing of skin images (button “Foto”) and vertical movement of the camera-illuminator system (button “Motors”). Direct cable connection between camera and computer ensures fast image transfer and processing, allowing to display the obtained skin spectral images and the related clinical criteria on system’s monitor in less than a minute, that is, still in presence of patient at the doctor’s office. All obtained high resolution skin spectral images are stored in the computer memory for further analysis, if necessary.

## 3 Processing of skin triple-wavelength image data

### 3.1 Skin chromophore distribution mapping

**Figure 4:**
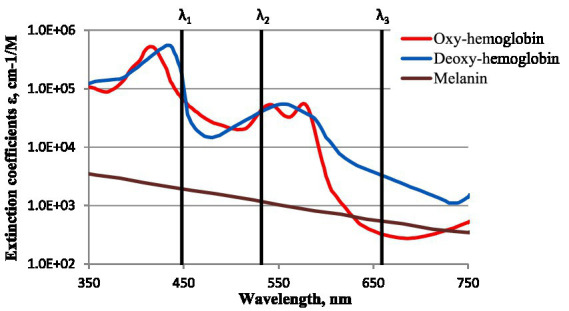
The absorption of three main skin chromophores (oxy-hemoglobin, deoxy-hemoglobin and melanin) at three fixed wavelengths (11).

The basic approaches for mapping the main skin chromophore variations in malformations by means of the 3WI technique are described in (11); general concept is illustrated at Figure 4. Let us suppose that RGB color image of a skin pathology spot, surrounded by healthy skin, is captured under illumination that comprises three equal intensity spectral lines at wavelengths λ_1_, λ_2_ and λ_3_ (the vertical lines in Figure 4). With respect to the spectral sensitivity of RGB image sensor and the crosstalk between its detection bands at the working wavelengths, three spectral line images are extracted from the color image data set by the technique described in (4). The RGB channel’s crosstalk is corrected using camera spectral sensitivities *S* at three working wavelengths [provided by manufacturer or measured directly (8)] and keeping in mind that the relative spectral sensitivities in frame of each detection channel remain constant. For R-channel, as example, it can be expressed as $S_{R_{\mathrm{jk}}}=S(R_{j})/S(R_{k})$ , where *j* and *k* denote two out of three wavelengths; the same applies to G- and B-channel.

The three-chromophore skin model suggests that the dominant absorbers in skin at any of the fixed wavelengths λ_j_ (j = 1, 2, 3) are oxy-hemoglobin (further abbreviated by *a*), deoxy-hemoglobin (*b*) and melanin (*c*) – see the crossings of their absorption curves with the vertical lines in Figure 4. If skin surface reflection is suppressed, variations in chromophore composition would lead to changes of the diffusely reflected light intensities at each of the fixed wavelengths. Such variations in the pathology region relative to healthy skin can be estimated by measuring reflected light intensities from equally sized regions of interest within the pathology (*I_j_*) and the adjacent healthy skin (*I_jo_*). The ratios *I*_j_ */ I*_jo_ at each pixel or pixel’s group of three spectral line images contain information on the concentration increase or decrease of all three regarded chromophores Δc_i_ (i = a, b, c), which can be further mapped over the whole image area.

The reflected intensity changes at three exploited wavelengths with different absorption can be represented in terms of the Beer–Lambert–Bouguer law (15):


$$\left\{ \begin{array}{c} \ln(\frac{I_{1}}{I_{01}})=-l_{1}(\Delta c_{a}\cdot \varepsilon_{a}(\lambda_{1})+\Delta c_{b}\cdot \varepsilon_{b}(\lambda_{1})+\Delta c_{c}\cdot \varepsilon_{c}(\lambda_{1}))\\ \ln(\frac{I_{2}}{I_{02}})=-l_{2}(\Delta c_{a}\cdot \varepsilon_{a}(\lambda_{2})+\Delta c_{b}\cdot \varepsilon_{b}(\lambda_{2})+\Delta c_{c}\cdot \varepsilon_{c}(\lambda_{2}))\\ \ln(\frac{I_{3}}{I_{03}})=-l_{3}(\Delta c_{a}\cdot \varepsilon_{a}(\lambda_{3})+\Delta c_{b}\cdot \varepsilon_{b}(\lambda_{3})+\Delta c_{c}\cdot \varepsilon_{c}(\lambda_{3})) \end{array}\right.$$ (1)

where ε_i_(λ_j_) - extinction coefficients of three regarded chromophores at three exploited wavelengths, *l_j_* – the mean path length of remitted photons in skin at a particular wavelength. Chromophore concentration increases or decreases at each image pixel (or selected group of pixels) are found by solving Equation 1 with abbreviated measured quantities *k_j_* = ln (*I*_j_*/I*_jo_):


$$\begin{array}{ll} \Delta c_{a} & =A_{1}\cdot k_{1}+A_{2}\cdot k_{2}+A_{3}\cdot k_{3}\\ \Delta c_{b} & =B_{1}\cdot k_{1}+B_{2}\cdot k_{2}+B_{3}\cdot k_{3}\\ \Delta c_{c} & =C_{1}\cdot k_{1}+C_{2}\cdot k_{2}+C_{3}\cdot k_{3} \end{array}$$ (2)

The coefficients *A_j_*, *B_j_* and *C_j_* in Equation 2 comprise numerical values of the corresponding chromophore extinction coefficients ε_i_(λ_j_) and the remitted photon path lengths *l_j_* at three exploited wavelengths. The *l_j_* values are estimated from our measured photon time-of-flight (TOF) in forearm skin [10 volunteers, Fitzpatrick photo-types II and III, 35 spectral/spatial combinations (16)]. Exact values of the remitted photon path length in skin of other body locations and for other photo-types may be different, but their wavelength-relations are similar. Unfortunately, the available measured data is very limited, while the published simulated photon path lengths appear less reliable as they hardly depend on the used anatomical model of skin.

**Figure 5:**
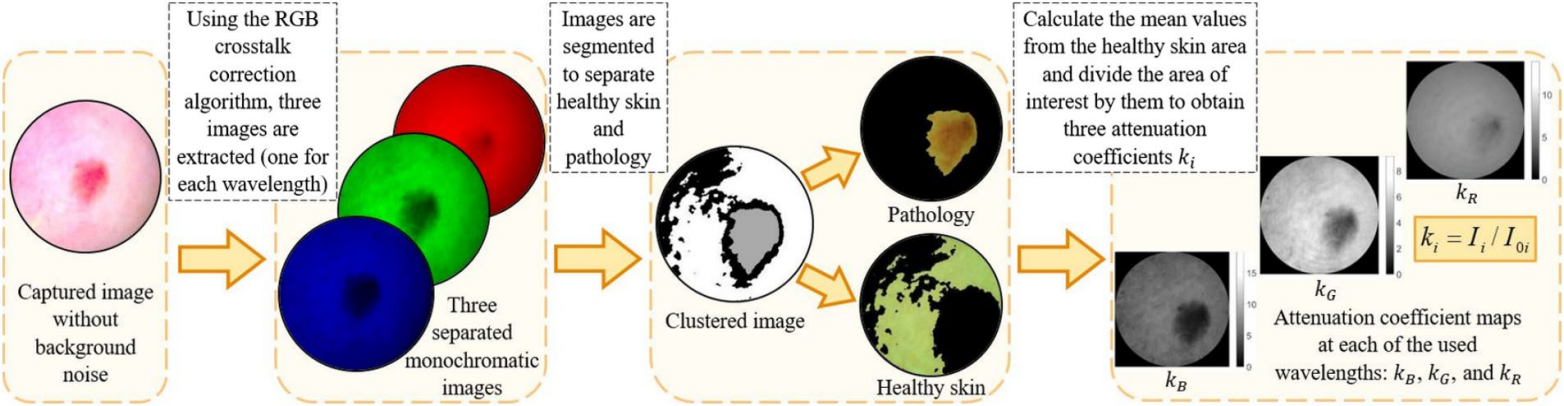
Three-wavelength image processing scheme of steps for obtaining three chromophore distribution maps (18).

The 3WI processing scheme is shown in Figure 5. The RGB image taken under three laser line illumination is split into three images – one for each exploited wavelength, applying the RGB crosstalk correction algorithm. Next, the images are segmented to separate the healthy skin from the skin malformation. From the segmented healthy skin, the average signal values at each of the illumination wavelengths ( $I_{0}$ ) are calculated; they are used as reference values when there is no additional absorption in the skin like in the pathology. The spectral images ( I ) are then divided by the reference values, yielding three attenuation coefficient maps ( $k(\lambda )=I/I_{0}$ ). These coefficient maps are further transformed into high-resolution (pixel size ~10 microns) distribution maps of chromophore concentration changes in the examined skin malformations.

### 3.2 Multi-lesion skin image processing

**Figure 6:**
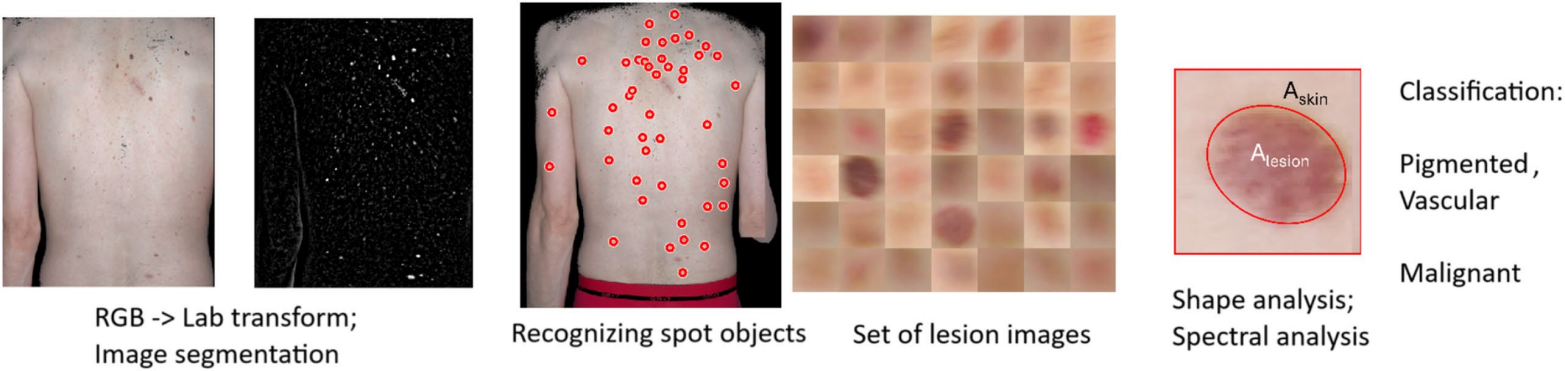
Lesion image processing steps to analyze large multi-lesion skin areas of human body (12).

The remotely captured large-area skin images usually contain more than one malformation. The developed 3WI processing algorithm (Figure 6) includes detecting all lesions of size ~1 mm^2^ (i.e., ~100 image pixels) or larger. At the first stage, a set of lesion images is created. Each image is then analyzed to find the shape and spectral properties of lesions. Finally, each lesion is classified as either pigmented or vascular. For this, the relative optical density (ROD, Equation 3) is used as a spectrally sensitive parameter:


$$\mathrm{ROD}=\log_{10}(I_{\text{skin}}/I_{\text{lesion}})$$ (3)

where $I_{\text{skin}}$ is the recorded reflected intensity of a corresponding spectral line (450 nm, 520 nm, or 638 nm) from a healthy skin area close to the lesion, and $I_{\text{lesion}}$ is the reflected intensity from the segmented lesion area of the same size as the healthy skin area.

To classify each detected lesion as pigmented or vascular, correlations between the ROD values at 638 nm (*r,* red) and at 520 nm (*g,* green) are exploited. Pigmented formations have higher ROD*_r_* values compared to those of vascular ones; the detected skin malformations are classified as presented in Equation 4:


$${\mathrm{ROD}}_{r}/{\mathrm{ROD}}_{g}[\begin{array}{c} >d\;\to \text{pigmented}\\ \leq d\;\to \text{vascular} \end{array}$$ (4)

where *d* is a discriminant threshold value for both types of lesions. The optimal discriminant value *d* can be found iteratively as described in (10).

**Figure 7:**
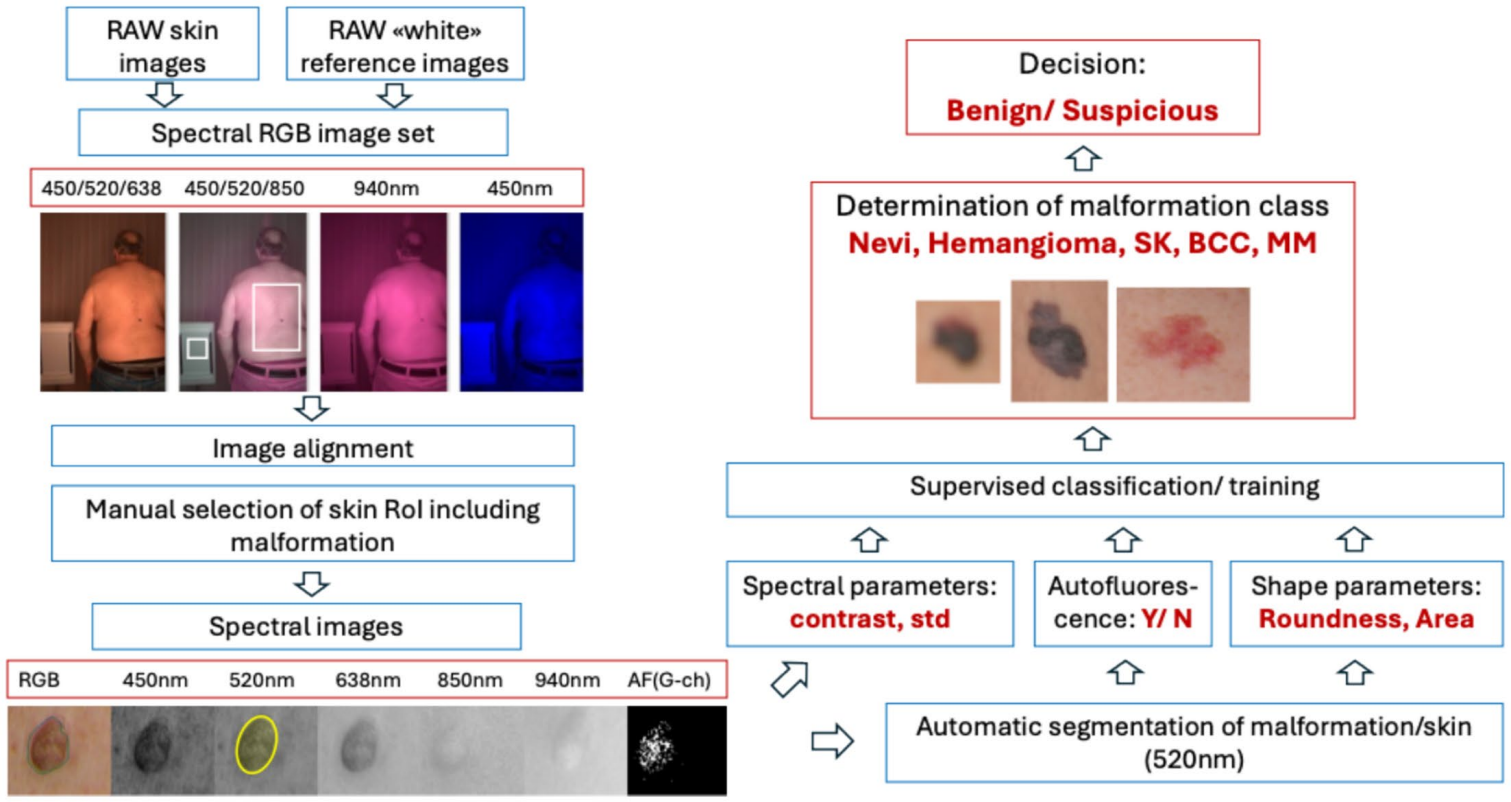
The scheme for obtaining and analysis of remotely captured skin malformation’s spectral images (13).

For comparative analysis of selected skin lesions represented in remotely captured visible and near-infrared (NIR) spectral images, a MATLAB (MathWorks, Natick, MA, United States) software solution as illustrated in Figure 7 can be applied. First, the RGB set is extracted from the registered RAW images of the white reference and skin. Next, RGB values are converted from the 16-bit to floating point precision and normalized as *I_RGB_ = I/I_ref_*, where *I_ref_* is white reference signal intensity. After manually selecting skin area with lesion, the malformation is automatically found and separated from surrounding skin using a binary mask which is calculated from 520 nm spectral image (by built-in MATLAB algorithms *imbinarize* and *regionprops*). Then spectral images are constructed using spectral unmixing operation *I_sp_ = A*^−1^ *I_RGB_*. The reflected spectral line intensities for each image pixel are found by solving linear matrix operations, as presented in (13).

As the next step, shape-related parameters – area S and roundness *R* - are calculated for each selected skin formation as $S=\sum \text{pixel}s_{\text{malf}}$ and $R=4\mathrm{\pi S}/P^{2}$ , where P – perimeter of the malformation. Besides, specific spectrally sensitive parameters for each malformation are calculated from the extracted spectral images:

- Contrast $C=\bar{I_{\text{skin}}}/\bar{I_{\text{malf}}}$ , where $\bar{I_{\text{malf}}}$ – median intensity of the malformation pixels, $\bar{I_{\text{skin}}}\;$ – median intensity of the surrounding healthy skin pixels (both with 520 nm segmentation mask);
- Empiric parameters, yielded from our clinical experience: $p_{450}=\frac{C_{638}\cdot C_{850}}{C_{450}}$ and $p_{520}=\frac{C_{638}\cdot C_{850}}{C_{520}}$ .

To explore the future potential of skin malformation’s automatic classification using AI approaches, the machine learning algorithm (MATLAB *classificationLearner* toolbox) was tested: each input in the training dataset of parameters was paired with a corresponding output label – the group of malformations (benign and malignant). Each skin formation was characterized by 19 parameters extracted from the set of its spectral images [including autofluorescence image at 450 nm illumination and standard deviations of pixel intensities inside and outside malformations at 4 wavelengths, see (13) for more details]. The model was trained with a validation scheme, to protect against overfitting by applying cross-validation; the data was set to five folds and the accuracy for each fold estimated. For data validation, linear support vector machines model (SVM) was chosen. This tool allows calculating validation confusion matrix, true-positive (TP), false-negative (FN), true-negative (TN) and false-positive rates (FP), as well as sensitivity TP/(TP + FN), specificity TN/(TN + FP), and accuracy (TP + TN)/n, where n – number of observations.

## 4 Clinical results

Clinical measurements with 3WI prototype devices were conducted at the University of Latvia, Riga Laser Plastic Clinic, and Oncology Center of Latvia on volunteers with their written consent. Inclusion criteria: age 18+, conscious condition, normal body temperature, ability to communicate in state language, non-pregnancy. The procedures were carried out in full compliance with the World Medical Association Declaration of Helsinki and approved by relevant ethics committees. The study design included standard diagnosis by a qualified dermatologist, followed by examination of selected malformations with a 3WI device and further spectral image analysis for defined skin pathology groups (e.g., nevi, hemangiomas, basal cell carcinomas). Main research goal was to identify spectral image features and parameters, specific for each of the examined groups and potentially suitable for automatic non-invasive skin diagnostics.

The typical skin tone in the general Latvian population corresponds to Fitzpatrick skin photo-type II. For example, in one of our studies (*n* = 164), 9.1% of volunteers had skin photo-type I, 90.3% had photo-type II, and 0.6% had photo-type III. Consequently, our clinical results are most applicable to skin photo-type II patients. For broader use, additional clinical trials involving a wider range of skin types are needed.

In our first public screening tests with the smartphone-based 3WI prototype, age of volunteers ranged from 20 to 88 years, with female/male ratio 3:1. In the potential malignancy measurement series with the remote whole-body 3WI device, 40- to 93-year-old volunteers participated, with nearly equal number of women and men.

Lesion sizes varied from a few millimeters to a few centimeters. Using the remote whole-body 3WI device, all lesions ≥1 mm across the volunteer’s bodies were captured. Smaller lesions contain fewer pixels and appear blurrier, but image quality remains sufficient for color analysis. These images also provide baseline location and appearance for future follow-up to assess size changes over time.

Measurements were taken from all body regions. In the public screening trials, most lesions were located on the anterior body (42%) and posterior body (36%); 10% were on the upper limbs and 8% on the lower limbs, and only 4% were on the head. In contrast, the potentially malignant lesions were located on the head (53%), anterior body (17%), posterior body (18%), upper limbs (7%), and lower limbs (5%).

### 4.1 Clinical validation of the smartphone-based 3WI prototype

The smartphone-based 3WI prototype described in section 2.1 was clinically validated on vascular and pigmented skin lesions (11). Nine vascular and pigmented skin lesions were examined in the first validation trial, including three pigmented nevi, three seborrheic keratosis, and three hemangiomas; all mentioned skin malformations were diagnosed by an experienced dermatologist using dermatoscope. The main goal of this trial was to check if the above-described hardware and software can provide physiologically feasible information on already diagnosed skin malformations in terms of increased/decreased contents of three regarded chromophores.

**Figure 8:**
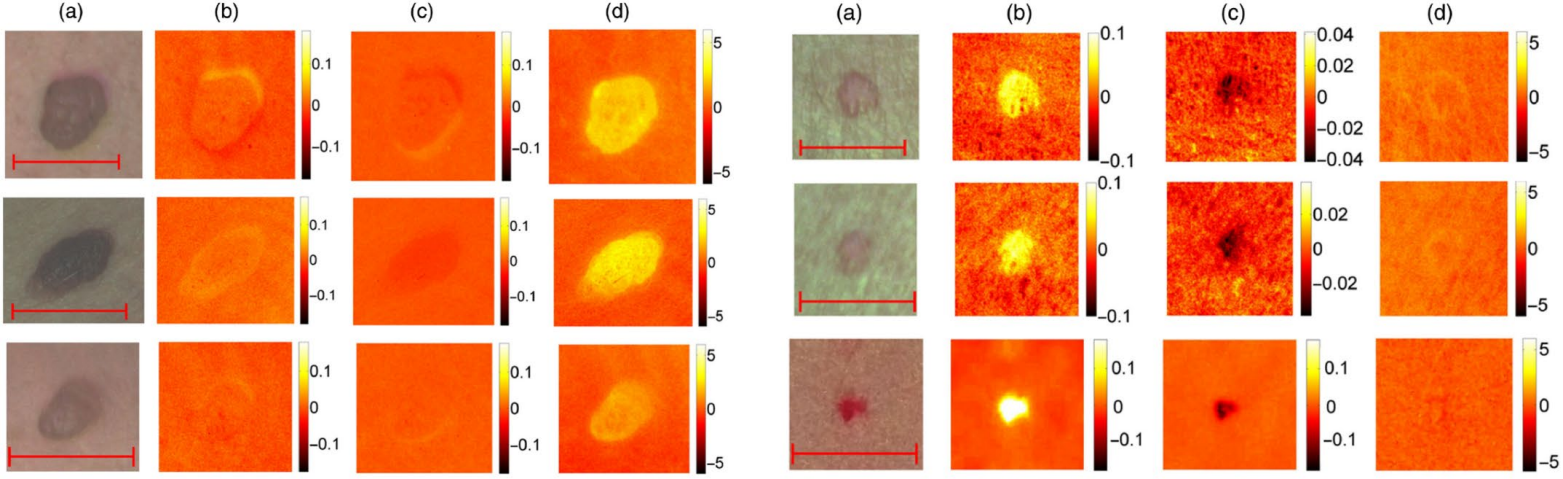
Colored clinical image of a lesion **(a**, scale bar 5 mm**)** and the corresponding maps representing chromophore concentration changes for three cases of: pigmented nevi (left) and hemangioma (right); **(b)** – oxy-hemoglobin, **(c)** – deoxy-hemoglobin, **(d)** – melanin. Units of the color scale – millimoles (11).

During procedure, the 3WI device was positioned with the lesion centered in the field of view, then a single snapshot image under 3WI was captured. After processing the clinical images, skin chromophore maps were constructed and changes of lesion’s chromophore content with respect to the adjacent healthy skin evaluated. Following the clinical models, pigmented skin lesions represent abnormal increase of epidermal melanin and the vascular lesions – increased supply of dermal arterial blood (rich of oxy-hemoglobin) to the superficial layers of skin. As expected, notable melanin content increase was observed in all cases of nevi and seborrheic keratosis. Increase of oxy-hemoglobin content and decrease of deoxy-hemoglobin was observed in all examined hemangiomas (vascular malformations). As illustration, images for three typical clinical cases related to each of the examined three pathologies are presented in Figure 8. The color photo of a malformation, taken under simultaneous three wavelength illumination (on the left – A), is compared with the obtained concentration distribution maps of oxy-hemoglobin (B), deoxy-hemoglobin (C) and melanin (D). All pathology data are related to the adjacent healthy skin so that only increased and decreased concentrations are quantified by colors. One can see how melanin content increases in nevi, without essential changes in the hemoglobin content. Similar responses were obtained from the seborrheic keratosis, but quite different chromophore composition changes were recorded for vascular hemangiomas (Figure 8, right) – melanin concentration in the pathology remained practically unchanged while the oxy-hemoglobin content notably increased and the deoxy-hemoglobin content decreased in comparison to healthy skin.

**Figure 9:**
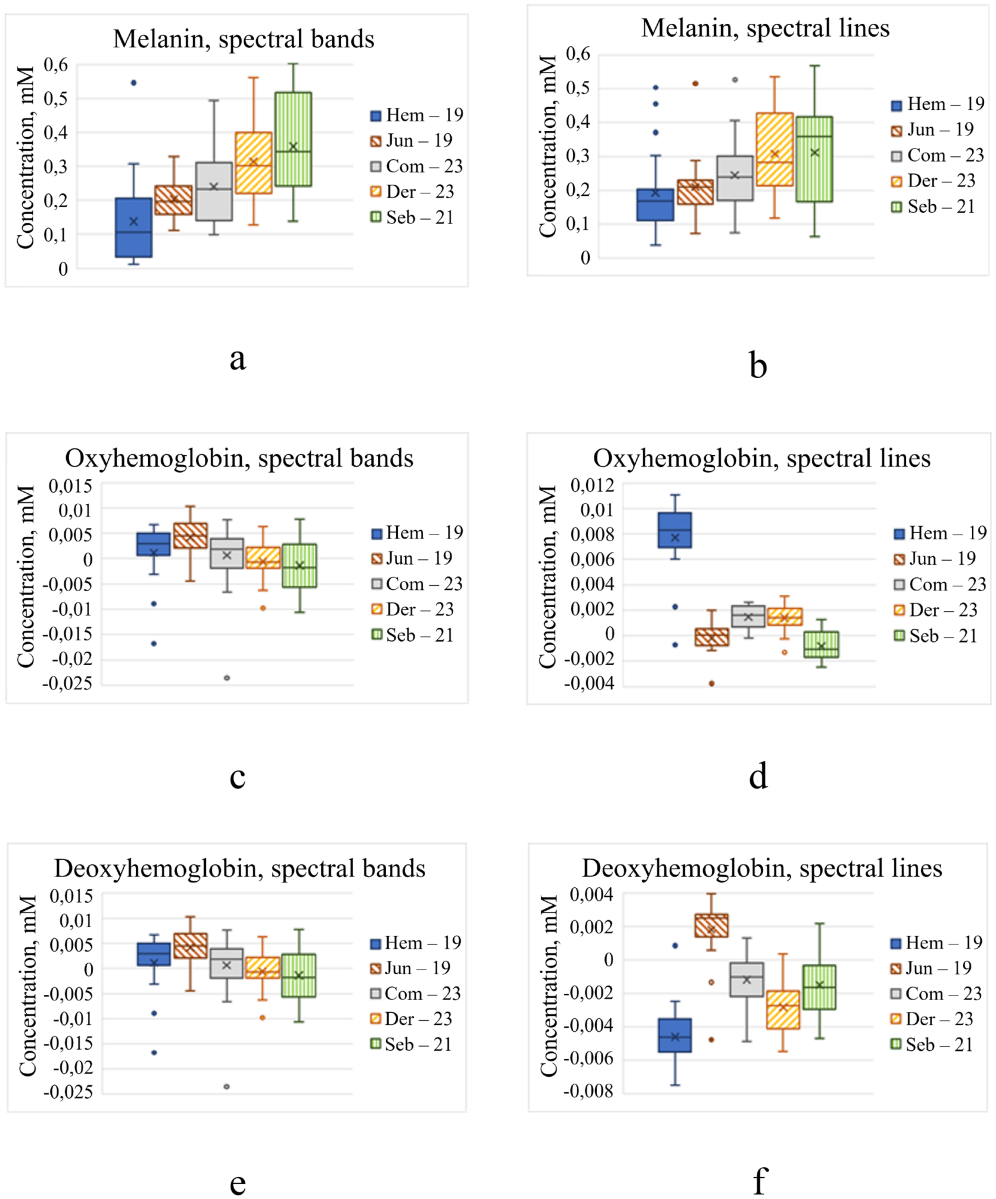
Comparison of the relative concentration changes of melanin **(a,b)**, oxyhemoglobin **(c,d)** and deoxyhemoglobin **(e,f)** in hemangiomas ( Hem ), junctional nevi ( Jun ), combined nevi ( Com ), dermal nevi ( Der ) and seborrheic keratoses ( Seb ), calculated for the spectral band **(a,c,e)** and the spectral line **(b,d,f)** illumination. Rectangles in graphs describe 25 to 75% values, vertical lines – standard deviations, crosses – mean values, horizontal lines – median values, circles – outliers (17).

The smartphone-based 3WI prototype was clinically validated in two other studies. In the second validation trial (17), the calculated chromophore composition of the same skin malformations was analyzed for two modalities of illumination. Chromophore maps obtained in similar geometry under sequential skin illumination by narrowband LEDs with emission spectral peaks at 460 nm, 535 nm, and 663 nm were compared with those obtained using the smartphone-based 3WI device; the same calculation algorithm was used in both cases. This was done to determine whether the laser-based modality should be retained or, if comparable results could be achieved, replaced by triple-LED illumination, which is less expensive and simpler to implement. The study included 21 hemangiomas (vascular lesions), 23 seborrheic keratoses, and 61 nevi (19 junctional, 23 dermal, and 19 compound). The results demonstrated essential differences in chromophore maps calculated from images captured at both illumination modes, as well as in the concentration changes of three main skin chromophores – see Figure 9 for illustration. For instance, oxy-hemoglobin content in hemangiomas does not differ much from the other skin malformations considered if the spectral band images are processed. The spectral line image processing, in contrast, clearly distinguishes hemangiomas. Furthermore, junctional nevi tend to show elevated deoxy-hemoglobin, allowing partial separation if the triple spectral line imaging technology is applied. The larger error bars arise primarily from substantial inter-lesion variability: nevi, for instance, can range from very lightly pigmented to dark brown, and seborrheic keratoses and hemangiomas likewise exhibit considerable heterogeneity. The reported parameters represent mean melanin, oxy-hemoglobin, and deoxy-hemoglobin concentrations averaged over the entire lesion area; consequently, lighter lesions yield lower mean melanin estimates, whereas darker lesions yield higher values.

Generally, the provided comparison confirms the risk that skin chromophore map calculations from conventional spectral band images, considering only the peak wavelengths of imaging bands, may lead to mistaken results. Image processing in such cases should involve integration over all wavelengths comprised at each spectral band of illumination, which is much more complicated and time-consuming. Clear advantages of the 3WI approach for skin chromophore mapping have been demonstrated by this study.

**Figure 10:**
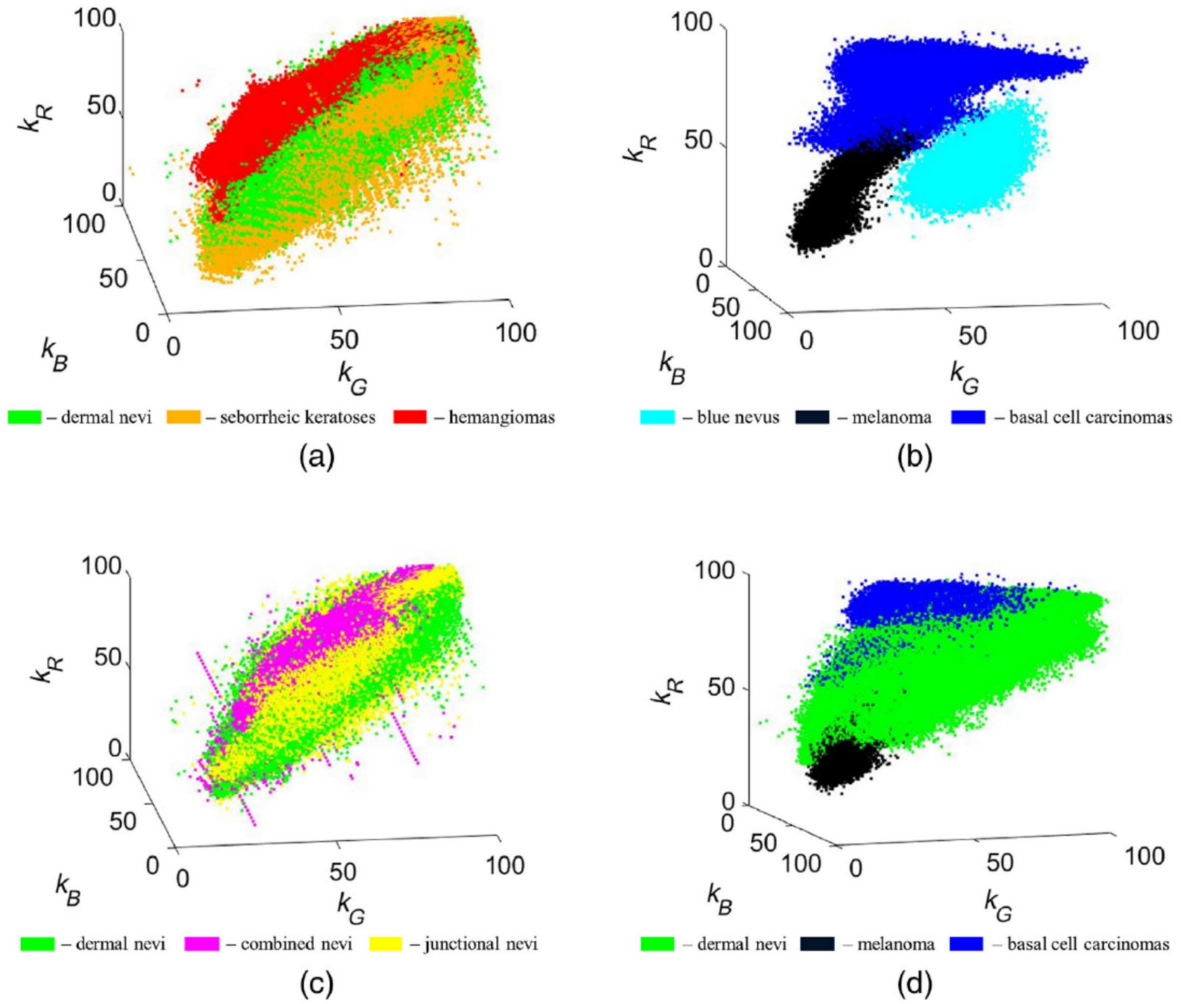
3D graphs of the three attenuation coefficients (in %) for different groups of skin lesions: dermal nevus, seborrheic keratosis and hemangioma **(a)**, blue nevus, melanoma and basal cell carcinoma **(b)**, dermal, combined and junctional nevus **(c)**, dermal nevus, melanoma and basal cell carcinoma **(d)** (18).

In the third validation trial, 3D-representation of the 3WI imaging data for improved skin diagnostics was proposed and tested (18). In total, 99 diagnosed skin pathologies were examined: 3 basal cell carcinomas, 27 dermal nevi, 12 hemangiomas, 16 combined nevi, 1 melanoma, 17 junctional nevi, 22 seborrheic keratoses and 1 blue nevi. The smartphone 3WI prototype device was used to measure the reflected intensity ratios - pathology *vs* healthy skin, $k_{j}$ - at the three working wavelengths *j*. The k-values for 450 nm are denoted as k_B_, those for 532 nm – as k_G_, and those for 659 nm – as k_R_. Each of them represents one of three coordinate axes in the 3D graphs shown in Figure 10. Every point in the graph represents one clinical data pixel signal value from a segmented pathology. Points are arranged more densely where pixel output signals have similar values, and they are more scattered where only few pixels have these values. Each cloud consists of ~ 6,000 to ~400,000 non-overlapping points.

The k-value clouds for dermal nevi, seborrheic keratoses and hemangiomas are compared in Figure 10a. Parts of them are non-overlapping, for example, the specific volume related to hemangiomas and that related to nevi can be easily distinguished. Even better separation between malformations can be observed in Figure 10b where spectral attenuations of two malignant pathologies – melanoma and basal cell carcinoma – are compared with those of a benign pathology – blue nevus. All three pathologies here can easily be distinguished; the $k_{R}$ values for blue nevus and melanoma are lower than those for basal cell carcinoma, while melanoma exhibits lower $k_{G}$ values than the blue nevus. Spectral attenuations of three different types of nevi - dermal, combined and junctional – are compared in Figure 10c; they form a compact cloud but still each type mainly covers a specific volume in the 3D space. Data for malignant pathologies (basal cell carcinoma and melanoma) and typical benign pathology (dermal nevus) are compared in Figure 10d. Again, some values of all three malformations are slightly overlapping but the $k_{R}$ values for nevi are clearly higher than those for melanoma and lower than those for basal cell carcinomas.

To summarize, the obtained 3D-graphs exhibit specific volume-shape features for each of the eight examined groups of skin pathologies. This kind of spectral image data representation may find further application in quantitative diagnostics of skin malformations, for example, for characterizing and identifying specific skin pathologies by the spatial location of the $k_{B}-k_{G}-k_{R}$ data points determined from the triple-wavelength spectral images.

### 4.2 Clinical validation of the remote whole-body 3WI device

Thirty dermatology patients with various skin malformations and 10 healthy volunteers participated in the first clinical validation of remote whole-body 3WI device, where altogether 4,185 pigmented and vascular skin malformations were detected and analyzed (12). The group of volunteers comprised 28 females and 12 males aged 20 to 73 years. A sub-group of patients with pigmented atypical lesions (as assessed by the dermatologist) was selected for further spectral analysis.

In terms of parameters ROD*_r_* and ROD*_g_*, linear regressions were found: ROD*_r_* = 0.52 *×* ROD*_g_ −* 0.02 for pigmented lesions and ROD*_r_ =* 0.1 × ROD*_g_* for vascular lesions. After the final iteration, optimal separation between 3,117 pigmented and 1,068 vascular formations was achieved, with the border between two types of lesions described by linear equation ROD*_r_ =* 0.18 × ROD*_g_*. Consequently, the malformation can be classified as pigmented if its ROD*_r_/*ROD*_g_ >* 0.18 and as vascular if ROD*_r_*/ROD*_g_ <* 0.18.

Another clinical validation trial of the remote whole-body 3WI prototype device was conducted at the Oncology Center of Latvia, focusing on the potential of visible and NIR contactless spectral imaging for detection of skin malignancies (13). A total of 60 patients participated in the study, including 31 women and 29 men, aged between 35 and 93 years. Patients were examined right after visiting experienced dermatologists who diagnosed their skin lesions with standard equipment. Triple-wavelength images of the patient’s skin areas with malformations were captured in a dark room, with sequential switching between spectral modalities of illumination: (i) combined 450/520/638 nm laser lines, (ii) combined 450/520/850 nm laser lines, (iii) each illumination line separately. The target skin area was in focus and well illuminated. All patient imaging data were anonymized and stored in a dedicated database for further analysis.

Histologically confirmed clinical diagnoses involved malignant melanoma (MM, *n* = 7) and basal cell carcinoma (BCC, *n* = 37), along with clinically confirmed 11 BCC, 84 nevi, 27 hemangiomas, 29 seborrheic keratoses (SK) and single cases of Bowen’s disease and blue nevus. In total, more than 400 spectral line images of different skin lesions were captured and 184 malformations analyzed.

**Figure 11:**
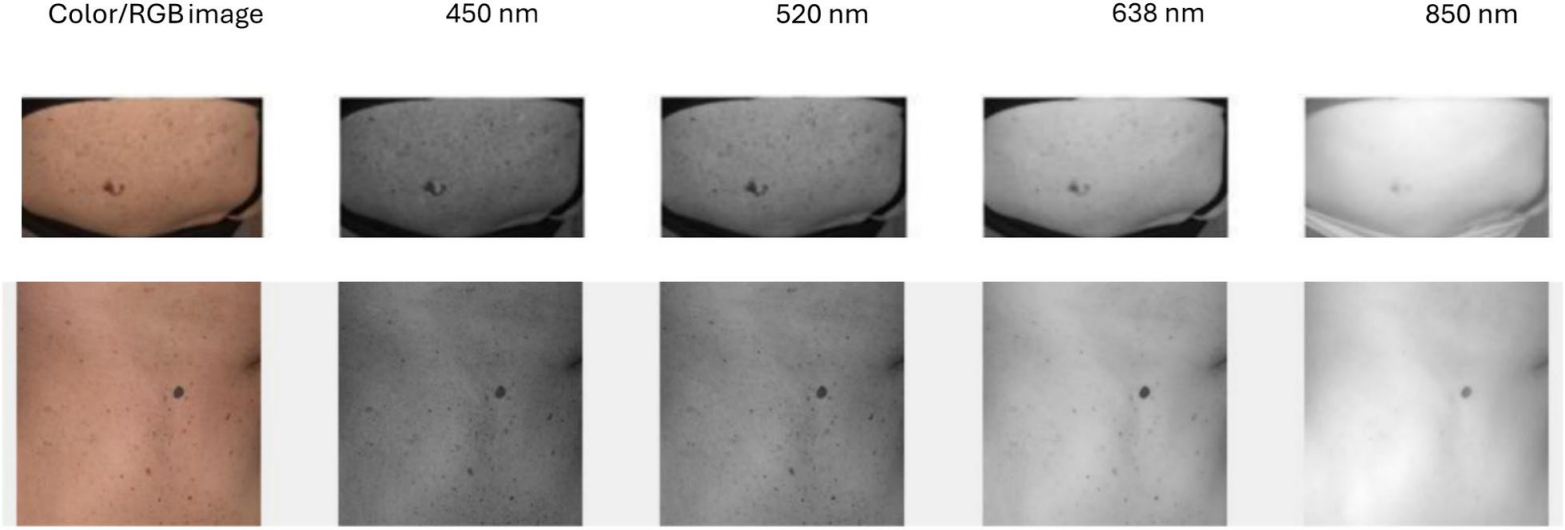
Comparison of spectral line images acquired from two cases of malignant melanoma surrounded by multiple nevi and photodamaged skin on the patients’ backs. While the visibility of the surrounding benign lesions progressively decreases with increasing wavelength, both melanomas remain clearly discernible in the 850 nm near-infrared image (NIR contrast persistence effect) (13).

The clinical utility of the NIR imaging is illustrated in Figure 11, which presents two cases of cutaneous melanoma surrounded by numerous benign nevi and photodamage-related skin lesions imaged at blue (450 nm), green (520 nm), red (650 nm), and near-infrared (850 nm) wavelengths. Most benign lesions that were clearly visible in the visible-spectrum images exhibited a progressive loss of contrast with increasing wavelength and became indistinguishable from the surrounding skin in the NIR images. In contrast, both melanomas remained clearly discernible at 850 nm, demonstrating a marked differential NIR contrast persistence between malignant and benign pigmented lesions.

Most benign lesions exhibited a wavelength-dependent attenuation of contrast and became indistinguishable in the NIR images, whereas melanomas remained clearly visible. This differential behavior, hereafter referred to as the “NIR contrast persistence effect,” may provide an additional diagnostic marker for melanoma detection.

**Figure 12:**
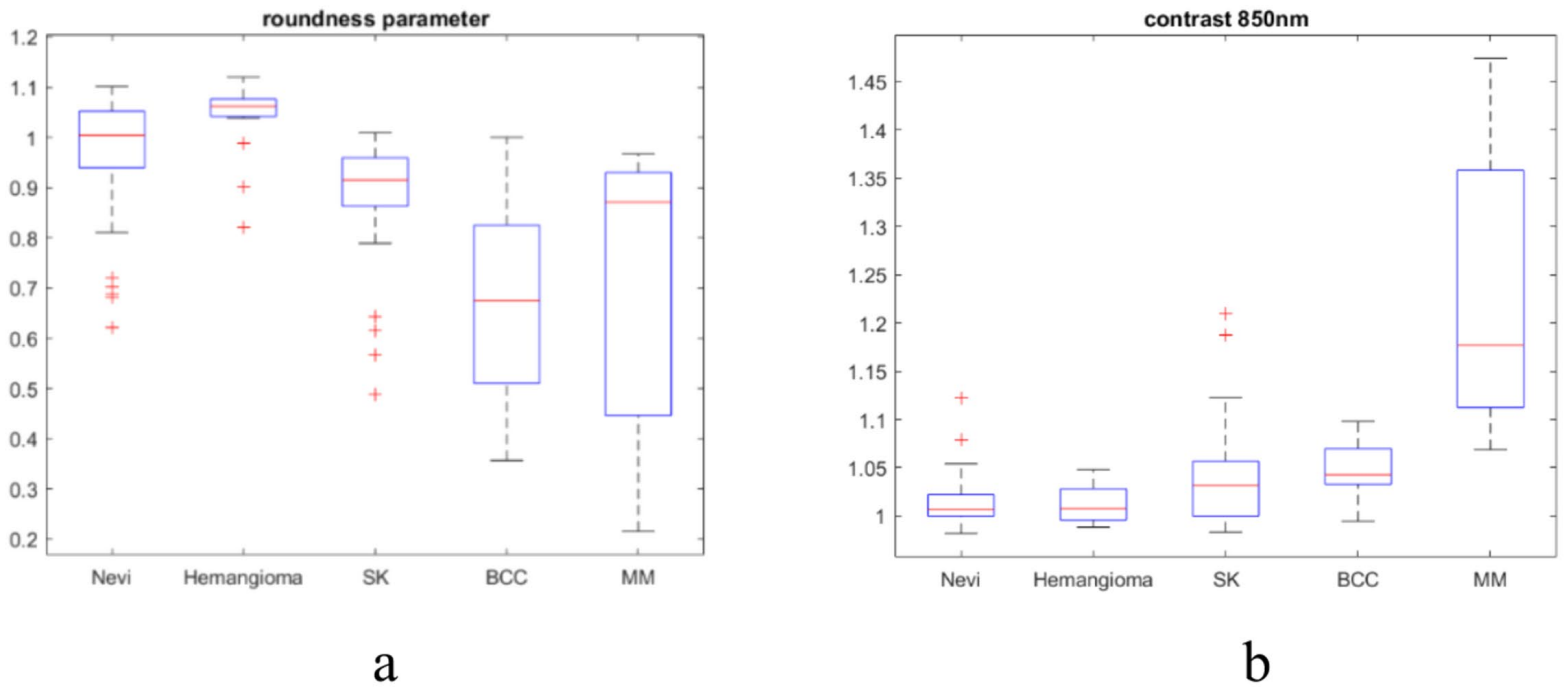
Statistical comparison of the roundness **(a)** and NIR-contrast **(b)** parameters for five classes of skin lesions: nevi, hemangiomas, seborrheic keratoses (SK), basal cell carcinomas (BCC) and malignant melanomas (MM) (13).

However, several benign lesions remained visible in the 850 nm NIR images, including hemangiomas, as well as a limited number of keratoses and nevi. The persistence of hemangiomas in NIR images is likely associated with the optical absorption properties of hemoglobin, whereas the visibility of certain keratoses and nevi may be related to increased lesion thickness and elevated melanin content, respectively. To distinguish them from the malignant skin formations, spectral image contrast *C* and two empiric parameters (*p_450_* and *p_520_*) were calculated. They demonstrated good selectivity for remote visible-NIR detection of skin malignancies. In Figure 12, the roundness parameters and contrasts in the 850 nm spectral images are compared for five selected classes of the examined malformations. In terms of roundness, both melanomas and basal cell carcinomas convincingly stand out, with values below 0.8 to characterize skin malignancies. Contrast in the 850 nm NIR-images (Figure 12b) appears a reliable melanoma marker, as melanomas exhibit the contrast values above 1.1 while basal cell carcinomas along with other NIR-detected malformations are characterized by lower NIR-contrast values.

**Figure 13:**
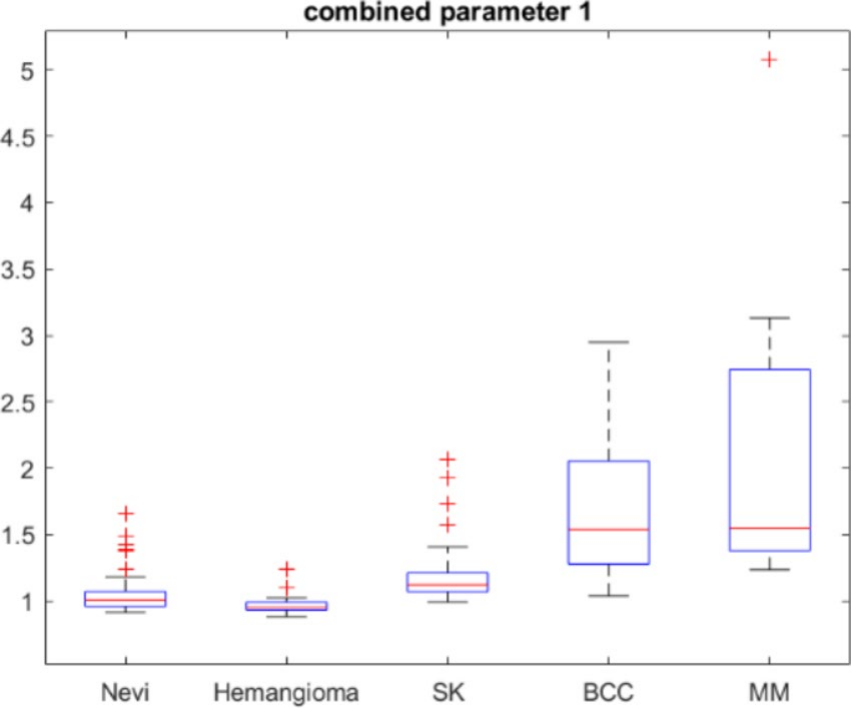
Combined parameter *C_850_/R* calculated for five classes of lesions: basal cell carcinoma (BCC) and malignant melanoma (MM) exhibit distinctly elevated values compared with the other lesion categories (13).

**Figure 14:**
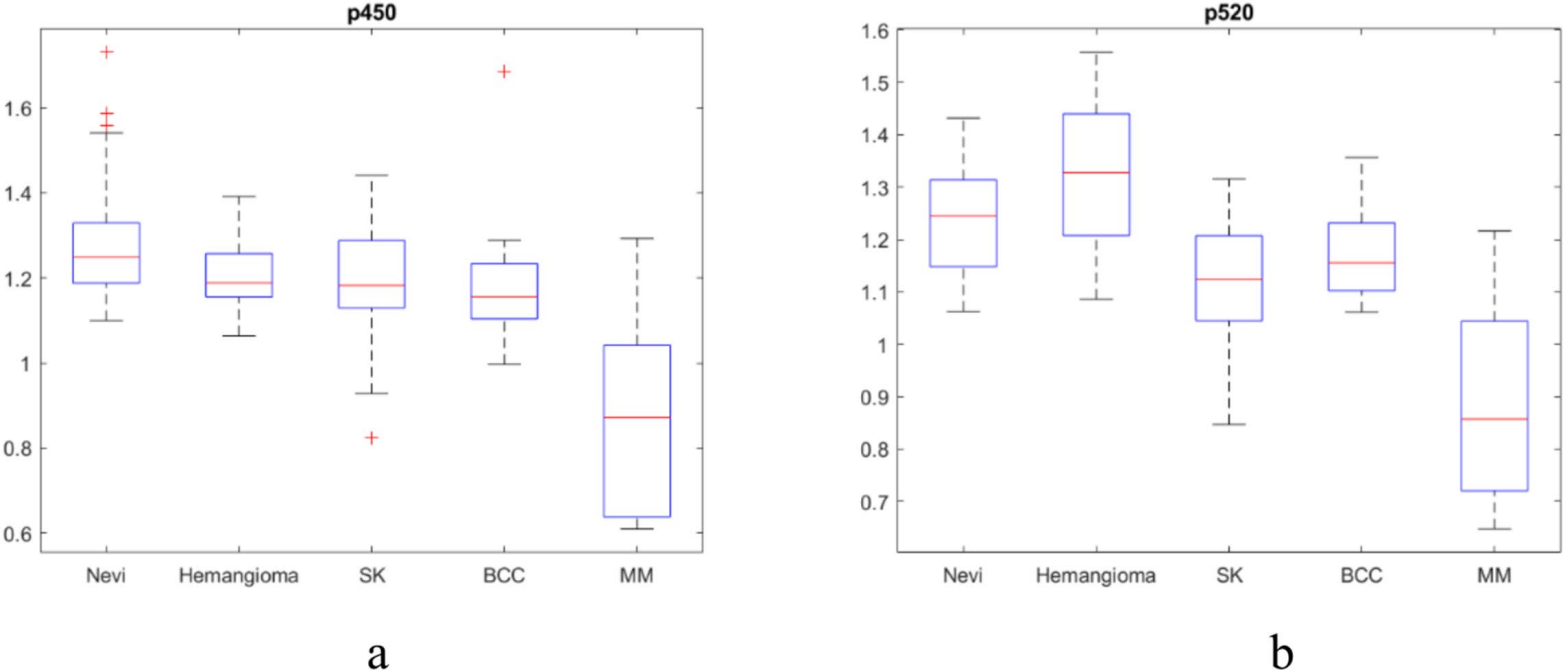
Values of the melanoma-specific parameters *p_450_* **(a)** and *p_520_* **(b)** for five classes of skin lesions: nevi, hemangiomas, seborrheic keratoses (SK), basal cell carcinomas (BCC) and malignant melanomas (MM) (13). MM exhibit distinctly lower values compared with the other lesion categories.

Furthermore, the NIR contrast–to-roundness ratio appears potentially useful for fast selection of skin malignances by single snapshot under double-wavelength 520 nm and 850 nm illumination - see Figure 13 where the *C_850_/R_520_* ratio value ~1.3 separates the benign and malignant skin formations. Additional selection of skin melanomas can be achieved by exploiting the empiric parameters *p_450_* and *p_520_* with the double-snapshot imaging technique – for example, first under visible RGB triple-line and then under NIR line illumination (wavelength combinations 450/520/638 nm and 850 nm, respectively), see Figure 14.

**Figure 15:**
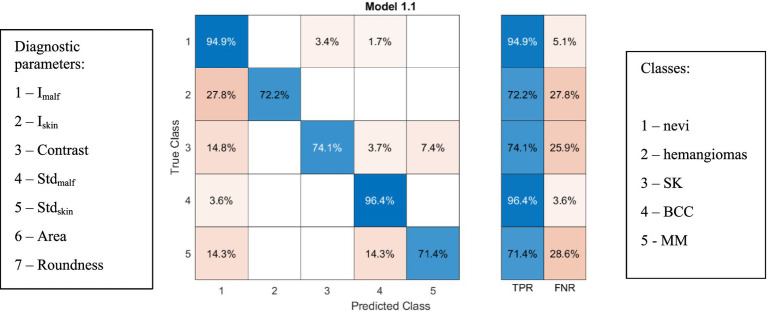
Results of the AI-based automatic classification of skin lesions using seven specific spectral image parameter sets captured at: 450 nm, 520 nm, 638 nm, and 850 nm (19 parameters for each lesion) (13).

Figure 15 presents the results of our AI-based calculations, testing the ability of AI for automatic malformation’s classification. One can see that the AI-prediction rate appears relatively high, reaching the true positive values in the range 71–96%. Nevi and basal cell carcinomas were well-recognized (true positive rate TPR 94.9 and 96.4%, respectively); as expected, AI precision critically depended on the number of used samples - that is why the calculated TPR for melanoma (*n* = 7) was the lowest in this study.

Finally, five groups of formations were re-arranged into two groups (184 observations): (1) benign, including nevi, hemangiomas and SK, and (2) malignant, including BCC and MM. From AI model calculations including all 19 shape and spectral parameters, we found the following statistical metrics: sensitivity = 0.89 (confidence interval 0.75–0.96), specificity = 0.99 (0.96–1.00), accuracy = 0.97. Similar values were obtained exploiting the criterion C_850_/R: sensitivity 0.886 (0.754–0.962), specificity (benign) 0.971 (0.928–0.992), and accuracy 0.951. Using specific combination of three parameters (C_850_/R_520_, p_450_, p_520_), the resulting malignance sensitivity was 0.77 (0.62–0.88), specificity 0.99 (0.95–1.00), and accuracy 0.93. When focusing on melanoma using the set of criteria C_850_, p_450_ and p_520_, the resulting sensitivity was 0.659 (0.501–0.795), specificity 0.950 (0.900–0.980), and accuracy 0.880.

## 5 Discussion

Recent progress in a relatively simple but informative and time-saving imaging modality for non-invasive skin diagnostics by conventional color cameras has been reviewed. To evaluate its suitability for routine clinical implementation, both technical and diagnostic aspects should be considered. From the technical point, the main challenges include selection of relevant triple-wavelength light source(s) and appropriate illumination designs that ensure obtaining high-quality set of skin spectral line images. Our studies confirmed suitability of compact diode laser combinations for triple wavelength emission, involving either separate laser modules (Figure 1) or single-unit RGB lasers (Figure 3). Specific bottleneck in both cases is the need for uniform illumination of the examined skin area simultaneously at all wavelengths, as the laser beams are highly directed and illuminate only small spots. In our studies, special light-diffusing arrangements have been designed, including solid state disc-shaped diffuser and side-emitting optical fiber loops, as illustrated above (Figures 1a, 3a). Equally important is proper processing of the obtained skin spectral image data to extract clinically valuable information from them; our solutions proved to be efficient for obtaining new or additional clinical information. As example, quantitative estimation of chromophore content changes in skin pathologies by a single snapshot of smartphone camera under appropriate illumination (Section 2.1) could be useful for diagnosis of cancer-suspicious skin formations. The 3D-representation of results obtained by the same equipment helps classifying several kinds of skin malformations (Figure 10). Automatic sorting of numerous pigmented and vascular skin lesions exhibited in large-area skin images can save doctor’s time if focusing only on pigmented or only on vascular lesions.

Dermoscopy significantly improves the accuracy of diagnosing melanoma and other skin cancers compared with naked-eye examination, thereby reducing unnecessary excisions (19). It reveals patterns and structures that are not visible to the naked eye, particularly when polarized dermoscopy is used. However, dermoscopy is operator-dependent, so less experienced clinicians may miss malignancies. It also has a limited field of view, typically ~1 cm in diameter. Examining every lesion on the entire body is time-consuming, and some lesions — particularly subtle amelanotic cancers — may be missed. Therefore, an automated system that flags potentially suspicious lesions from large skin areas could support faster and more comprehensive screening in a matter of minutes.

Invisible dermoscopy technology is still evolving, and UV and sub-UV dermoscopy represent recent advances over standard dermatoscopes (20). These noninvasive imaging methods can improve evaluation of neoplastic and inflammatory dermatoses by revealing features not visible under conventional light, helping optimize melanoma safety margins, identify prior biopsy sites, and narrow the differential diagnosis (e.g., erythrasma vs. psoriasis) through characteristic fluorescence patterns (21). However, limitations remain, including difficulty distinguishing melanin from hemoglobin in reflectance images and reduced accuracy due to uneven skin, pressure artifacts, or interference from topical products (sunscreens/cosmetics). Remote NIR imaging, as shown in this paper, may efficiently contribute to further developments of the invisible dermoscopy.

Orange light (581–600 nm) is also used to enhance visualization of pigmented regions and deeper subsurface structures by penetrating approximately 1–2 mm into the skin; because melanin absorption decreases above 570 nm, this spectral range can improve assessment of deeply pigmented areas compared with blue-tinted white light, supporting earlier skin cancer detection (22). Overall, these developments show that while dermoscopy is effective and widely used, there is still significant potential to improve diagnostic accuracy and reduce reliance on extensive operator expertise by making key features easier and more consistently visible, which could enable new methods that help clinicians identify diagnostic signs more efficiently, improve malignant lesion detection, and reduce unnecessary biopsies.

Conventional multispectral and hyperspectral imaging are conceptually similar to our approach but typically use more spectral bands. Both enhance contrast for melanin, hemoglobin/oxygenation and water, enabling applications such as lesion detection and monitoring of inflammation, wounds, and pigmentary disorders (23). Importantly, diagnostic performance depends on wavelength selection: UV/near-UV (≈365–405 nm) can capture autofluorescence for tumor margin assessment (24), visible wavelengths (≈450–600 nm) enable superficial melanin mapping (~470 nm) and vascular/perfusion evaluation via hemoglobin absorption peaks (≈525–550 nm) (25), and NIR (≈850–950 nm) offers deeper penetration and may aid differentiation because benign nevi often lose contrast near 940–950 nm whereas melanomas may remain detectable (5). As shown in our study, 850 nm NIR-imaging is equally efficient from this point. Existing commercial systems (e.g., MelaFind, SIAscope) generally provide high sensitivity but limited specificity, increasing false positives and unnecessary biopsies, and many function as “black-box” tools, limiting their role to adjunctive support (5). Future advances will likely come from multimodal, AI-assisted and miniaturized (e.g., smartphone-based) implementations that deliver interpretable, real-time, and remote assessment, contingent on improved specificity and validation in large multi-center trials. In this context, selecting an appropriate wavelength combination is key for targeting different skin features. If compared with conventional MSI/HSI, our proposed one−/two-snapshot method is faster, produces smaller files, and avoids band-to-band misalignment due to motion artifacts.

Smartphone-based dermatology screening is inexpensive and widely accessible for rapid imaging and self-monitoring, especially when combined with AI triage (26). In routine use, however, image quality and reproducibility are limited by uncontrolled and often non-uniform ambient illumination, automatic in-camera processing (white balance/HDR/sharpening/compression), differences between phone sensors/optics, and user-dependent factors such as distance, focus, and motion, leading to artifacts and domain shift. 3WI approach mitigates several of these limitations by using controlled built-in illumination that reduces ambient-light variability, fixed acquisition geometry (including defined working distance), and standardized capture settings via dedicated app/pro camera modes, thereby improving consistency across measurements.

We believe that the recently developed visible and NIR triple-wavelength remote skin imaging technique (13) can efficiently contribute to fast detection and screening of skin cancers. Melanomas and ulcerated basal cell carcinomas, due to their deeper invasion and higher blood content, are well-exposed in the NIR spectral images where most of the benign lesions disappear (Figure 11). To sort out skin malignancies, coincidence of two clinical criteria – roundness R (Figure 12a) and the ratio C_850_/R (Figure 13) can be exploited: R < 0.8 and C_850_/R > 1.3. Melanomas might be additionally identified by matching the critical values of three clinical criteria: C_850_ > 1.1, *p*_450_ < 1, and *p*_520_ < 1 (Figures 12b, 14). Along with the patient-friendly noncontact operation mode, advantage of the proposed methodology is short duration of the diagnostic procedure - remote single- or double-snapshot image capturing takes less than a second and the image processing time for automated malignancy evaluation typically is less than a minute.

As for the limitations of 3WI method, co-localized or overlapping malformations are difficult to separate using the current approach. Imaging on curved body regions (abdomen/flanks) is problematic due to nonuniform illumination, shadowing, and oblique viewing, which can distort lesion morphology and affect features such as roundness. Even if acquiring images from multiple views (front/back/sides), some distortion remains. A further limitation is that the developed diagnostic prototypes are highly custom-built; scaling to manufacturing will likely require redesign using standardized components. In addition, substantially larger datasets are needed, particularly for clinically suspicious lesions with histopathology as the reference standard.

Concerning the potential future developments, our smartphone-based design (Figure 1) could be further improved using smaller and lighter components, including a compact RGB laser module instead of six single-wavelength laser modules. The current version of remote triple-wavelength imager (Figure 2a) appears too bulky for routine non-contact skin cancer screening. To be more relevant, its design could be further developed as a portable device comprising high resolution black-and-white camera and a lighting unit ensuring fast sequential multi-LED illumination in the blue, green, red and NIR spectral bands. Future versions should capture more simultaneous multi-angle images and incorporate geometric corrections to improve detection on hard-to-image surfaces and to better estimate lesion roundness and lesion-to-skin contrast. Only pilot proof-of-concept studies of the 3WI approach have been performed so far, so more clinical data should be collected to better validate the proposed clinical criteria. Expanding the number of measured cases would enable more robust validation of image processing algorithms, refinement of model performance, and identification of the most informative parameters for lesion differentiation. Development of advanced software ensuring faster extraction of the visible NIR spectral images of skin malformations and calculation of appropriate malignancy criteria is a future task, as well. Summary.

An advanced imaging modality for assessment of skin malformations was presented and discussed in this paper, including technical solutions for equipment design and clinical data obtained by two experimental diagnostic prototype devices. The clinical potential of this approach includes ability for 2D- and 3D-mapping of chromophore content changes in skin pathologies, fast sorting of numerous pigmented and vascular malformations, and detection of skin malignancies in a non-contact mode. Further improvements in technical solutions and collection of more clinical data will facilitate implementation of triple-wavelength imaging as a routine diagnostic tool for dermatologists.

## Data Availability

The datasets presented in this study can be found in online repositories. The names of the repository/repositories and accession number(s) can be found at: https://dv.dataverse.lv.doi:10.71782/DATA/J0RMKK, DATAVERSE.
